# Recent advances in SERS-based bioanalytical applications: live cell imaging

**DOI:** 10.1515/nanoph-2023-0362

**Published:** 2024-03-06

**Authors:** Dong-Kwon Lim, Panangattukara Prabhakaran Praveen Kumar

**Affiliations:** KU-KIST Graduate School of Converging Science and Technology, 34973 Korea University, 145 Anam-ro, Seongbuk-gu, Seoul 02841, Republic of Korea; Department of Integrative Energy Engineering, College of Engineering, Korea University, 145 Anam-ro, Seongbuk-gu, Seoul 02841, Republic of Korea; Brain Research Institute, Korea Institute of Science and Technology, 5 Hwarang-ro 14-gil, Seongbuk-gu, Seoul 02792, Republic of Korea

**Keywords:** Raman scattering, surface-enhanced Raman scattering, live cellular imaging, Stokes-scattering, molecular signal, single cell analysis

## Abstract

Raman scattering can provide information on molecular fingerprints, which have been widely applied in various fields of material science and nanobiotechnology. Notably, low interference with water molecules in obtaining the Raman spectra between 500 and 2000 cm^−1^ made it a powerful spectroscopic tool in biology, such as imaging and signaling for a living cell. To be a robust tool for cell biology, the performance of obtaining molecular-specific information with high sensitivity, high resolution in real time, and without inducing cell damage is strongly required. The conventional fluorescence-based method has been suffered from the rapid photobleaching of organic fluorophores and the lack of molecular information. In contrast, Raman scattering is a promising spectroscopic tool to acquire cellular information, and the extremely low signal intensity of Raman scattering could be amplified by incorporating the plasmonic nanomaterials. Along with the fundamental research focus on surface-enhanced Raman scattering (SERS), the practical approaches of SERS for cellular imaging as a new tool for drug screening and monitoring cellular signals have been extensively explored based on new optical setups and new designing strategies for the nanostructures. Diverse nanostructure and surface chemistry for targeting or sensing have been played pivotal roles in acquiring cellular information and high resolution cell imaging. In this regard, this review focused on the recent advances of SERS-based technologies for a live cell imaging investigated such as potential drug screening, signaling for chemicals or biomolecules in cell, *in situ* sensing, and high spatiotemporal resolution.

## 1 Introduction

Raman scattering is an inelastic scattering of light, which provides the molecular information of analytes without interfering significantly with water molecules. Since the OH stretching mode in water is dominantly observed between 3000 and 3600 cm^−1^, most of the molecules in the cell showed a Raman shift between 500 and 2000 cm^−1^ [1]. Unlikely a fluorescence counterpart, a molecular spectrum of Raman scattering is not subject to photobleaching [2], [3]. In addition, the narrow spectral feature of Raman scattering with full width at half maximum enabled the achievement of highly informative chemical imaging of cells. There are diverse chemical components as a cell constituent, and the chemical state is dynamically changing with time, which accelerates the advance of new spectroscopic methods [4].

Currently, fluorescence-based methods are indispensable tools for fundamental research activity in life science as well as practical applications in the clinic [5]. It made many contributions to diverse fields of science. Determining the localization and dynamics of biomolecules such as DNA, RNA, and protein in a live cell is possible. The interaction between proteins of interest in a live cell is likely monitored by the fusion or dissociation of engineered proteins [6], [7], [8]. Many chemical fluorophores were designed to identify specific organelles inside a cell and measure the activity of a protein of interest. The specific structural feature of structures in a live cell could be visualized using super-resolution microscopy [5], [9]. However, very rapid photobleaching and limited molecular information are regarded as significant drawbacks of the fluorescence-based method [10].

In contrast to fluorescence-based spectroscopy, Raman spectroscopy is not commonly utilized as a practical tool despite a long development history [3]. This is mainly because of extremely weak signal intensity. The first finding of enhanced Raman signal intensity on Ag electrode surface by Fleischmann et al. [11], then interpreted the phenomena correctly in Refs. [12], [13], and the report of single-molecule sensitivity in Refs. [14], [15], ignited an extensive research focus on a fundamental understanding of the phenomena called surface-enhanced Raman scattering (SERS) [3]. Now the plasmon-assisted enhancement is relatively well understood. The localized surface plasmon resonance of plasmonic nanoparticles generated an enhanced electromagnetic field, and the molecules in this field showed a giant signal amplification. The two different mechanisms, such as electromagnetic (EM) field enhancement and charge transfer mechanism, are believed to be the origin of signal enhancement [3], [13], [16].

With an advance in the fundamentals of SERS, many new nanostructures and diverse strategies have been explored to utilize the SERS phenomena for analytical methods of small molecules or biomolecules such as DNA or protein [3], [17]. Typically, 2-dimensional substrates with array of nanostructures, such as sharp edge or nanogap, were proven to be useful for obtaining quantitative and sensitive analysis of small molecules [18], [19]. In contrast, nanoparticles dispersed in a solution can be used in a different form of application than a 2-dimensional substrate array. For example, nanoparticles with amplified Raman signal intensity can be used as a labeling material to detect target proteins [20], [21]. In addition, it can be used to detect various signal changes occurring in cells by delivering nanoparticles into cells, such as pH, redox potential, metabolites, or structural changes [4], [22].

In the early stage of this area, Nabiev et al. [23] proposed a method for cell imaging and intracellular Raman signal acquisition using SERS [23]. The interaction between the nuclei of living cancer cells and doxorubicin was studied using silver nanoparticles. In 2002, Kneipp et al. proposed a method for identifying Raman signals of phenylalanine and DNA in cells using gold nanoparticles [24]. Although it was useful to obtain a spectroscopic signal from a cell, the quality of single cell image was not comparable to that of fluorescence in view of spatiotemporal resolution. But, because of the strong potential for diverse bioanalytical applications, various studies for new applications have been conducted on using SERS for cell imaging. For example, metabolite monitoring, endogenous biomolecular imaging, molecular dynamics, cell surface investigation, and studies of receptor-ligand binding interactions have been exploited by incorporating a new plasmonic nanostructure, surface chemistry, and a new optical instrument.

In this review, the recent advances of SERS-based cellular imaging technology for diverse applications published within 10 years will be focused on, which can provide a comprehensive understanding of current challenges and future directions in adopting SERS technology for bioanalytical applications.

## 2 Label-free cellular imaging with Raman scattering

An ideal method to obtain intracellular information will be label-free chemical imaging. Even in the case of the fluorescence-based method, it has strongly relied on the use of exogenous fluorophores such as organic fluorescent molecules [10]. Using endogenous fluorophores such as fluorescent proteins (i.e., green, or yellow proteins) expressed by gene engineering could be an innovative tool in cell biology [6], [7]. In theory, Raman scattering can obtain spectroscopic information on all molecules with a polarizability change. It is possible to obtain a live cell Raman image based on the Raman peaks in the obtained spectrum [25]. In addition, a wide range of excitation wavelengths from UV to near-infrared (NIR) is possible to apply for Raman spectrum acquisition [26]. Using NIR wavelength for excitation is greatly beneficial for cell imaging because of the reduced autofluorescence background because of the off-resonance effect of NIR with biological components [26], [27].

**Figure 1:**
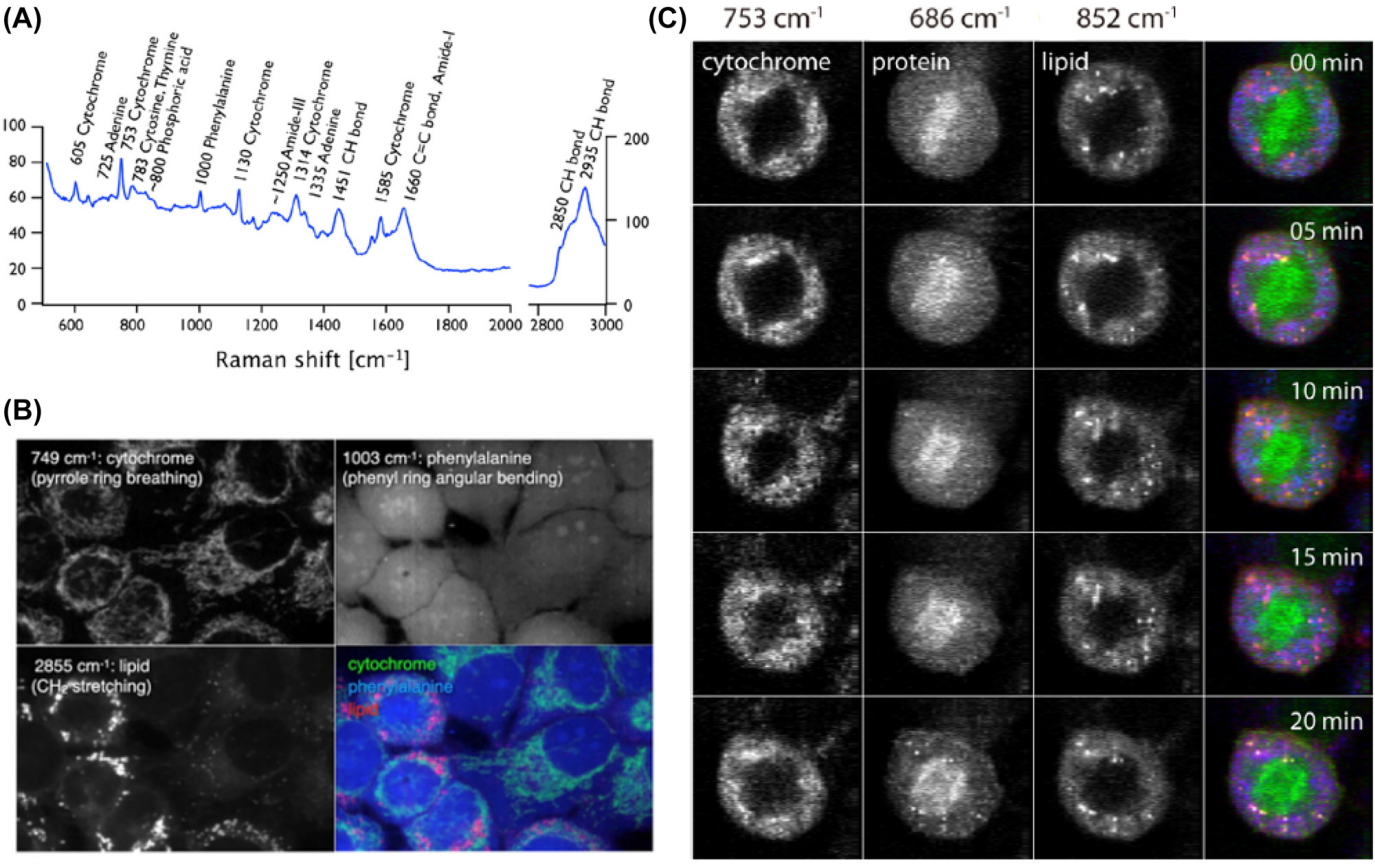
Representative Raman spectrum and images for the cellular components. (A) Raman spectrum obtained from cytosol in a live HeLa cell (excitation wavelength 532 nm). (B) Raman images reconstructed by selecting 749 cm^−1^ (mostly cytochrome), 1003 cm^−1^ (mostly phenylalanine), and 2855 cm^−1^ (mostly lipid). (C) Time-lapse single-cell Raman images of a HeLa cell during mitosis. Reprint permission was obtained for references [28], [29].

As shown in Figure 1A, a relatively complex Raman spectrum could be obtained from the cell [28]. Because of complex chemical substance inside cell, the Raman spectrum is typically complex, and also the spectral feature can be varied with time, and position of signal acquisition. However, some representative Raman peak could be obtained repeatedly. For example, the peak at 1451 cm^−1^ is the bending vibration mode of CH_2_ abundant in the hydrocarbon in cells (lipid molecules), and the Raman peak at 1660 cm^−1^ is the carbonyl vibration mode in the Amide-I of a peptide. Among these peaks, one can select a specific Raman peak of interest to construct the whole cell images, as shown in Figure 1B [29]. The Raman peak at 729 cm^−1^ is the pyrrole ring breathing mode in the cytochrome that existed in mitochondria, the Raman peak at 1003 cm^−1^ is the phenyl ring angular bending of phenylalanine abundant in protein, and the Raman peak at 2855 cm^−1^ is CH_2_ stretching mode of lipid could be seen mostly in the cell membrane, which can produce a whole cell image. These cell images were obtained with 532 nm wavelength excitation, 156 × 209 pixels, and the acquisition time was about 14 min [28]. The number of pixels can significantly affect the imaging quality and acquisition time. As shown in Figure 1C, it is Raman images of single Hela cells during mitosis. The pixels of each image were 48 × 161, and the acquisition time was about 3 min, which enabled to monitor of the changes in protein distribution every 5 min [28]. The imaging quality and overall acquisition time could be further improved with line-scanning confocal Raman microscopy [30].

As discussed, label-free Raman imaging is a quite useful method in obtaining a change of information during the intracellular process. However, the long acquisition time (several minute or hours, depending on the imaging quality) allows us only to observe the biological changes with relatively slow temporal changes [28]. Another limitation in the label-free Raman scattering for cell imaging is the strong autofluorescence background overlaps with that of Raman scattering. To address these issues, incorporating a nonlinear optical process (i.e., stimulated Raman scattering, coherent anti-Stokes Raman scattering, or second harmonic generation) is one of the development directions [29], [31]. Incorporating the new optical process can improve the temporal resolution, but the limited information of these nonlinear processes can be one of the intrinsic limitations. The use of exogenous small Raman tag molecules could avoid spectra overlap with the intracellular molecules, which can help to achieve an accurate location of molecules of interest [32], [33], [34].

## 3 Intracellular imaging with surface-enhanced Raman scattering (SERS)

In contrast to normal Raman scattering, SERS requires the use of plasmonic nanoparticles, typically gold or silver nanoparticles. Because of the greatly enhanced signal intensity of SERS, it is possible to reduce the exposure time per pixel greatly. Accordingly, the imaging time also could be greatly decreased, enabling real time monitoring of molecular signal changes. As a result, the improved temporal resolution is also greatly beneficial in improving spatial resolution by increasing the number of pixels in the field of view. In this section, the recent advances of SERS-based live cell imaging for various fields of application are discussed in detail.

### 3.1 SERS in a live cell for potential drug screening

In the development process of a drug candidate, monitoring the distribution of potential drug molecules in a live cell and understanding the dynamics of drug molecules in the targeted subcellular organelles is paramount [35], [36]. For potential drug screening or monitoring the dynamics of drug molecules in a cell, the uptake of plasmonic nanoparticles is required inside a cell in advance or simultaneously. An extremely low concentration of drug molecules and subtle changes in molecular signals could be observed by the signal enhancement of plasmonic nanoparticles.

Understanding the endo- and exocytic pathways related to endosomes or lysosomes is an important first step in the development of the basic science of cell biology and future medicine [37]. Kneipp et al. demonstrated that the use of gold nanoparticles (AuNPs) with size (30–50 nm) could identify the chemical information of endosomes in a live macrophage cell. Otherwise, it is required for the fractionation and purification of endosomes to study [24], [38].

**Figure 2:**
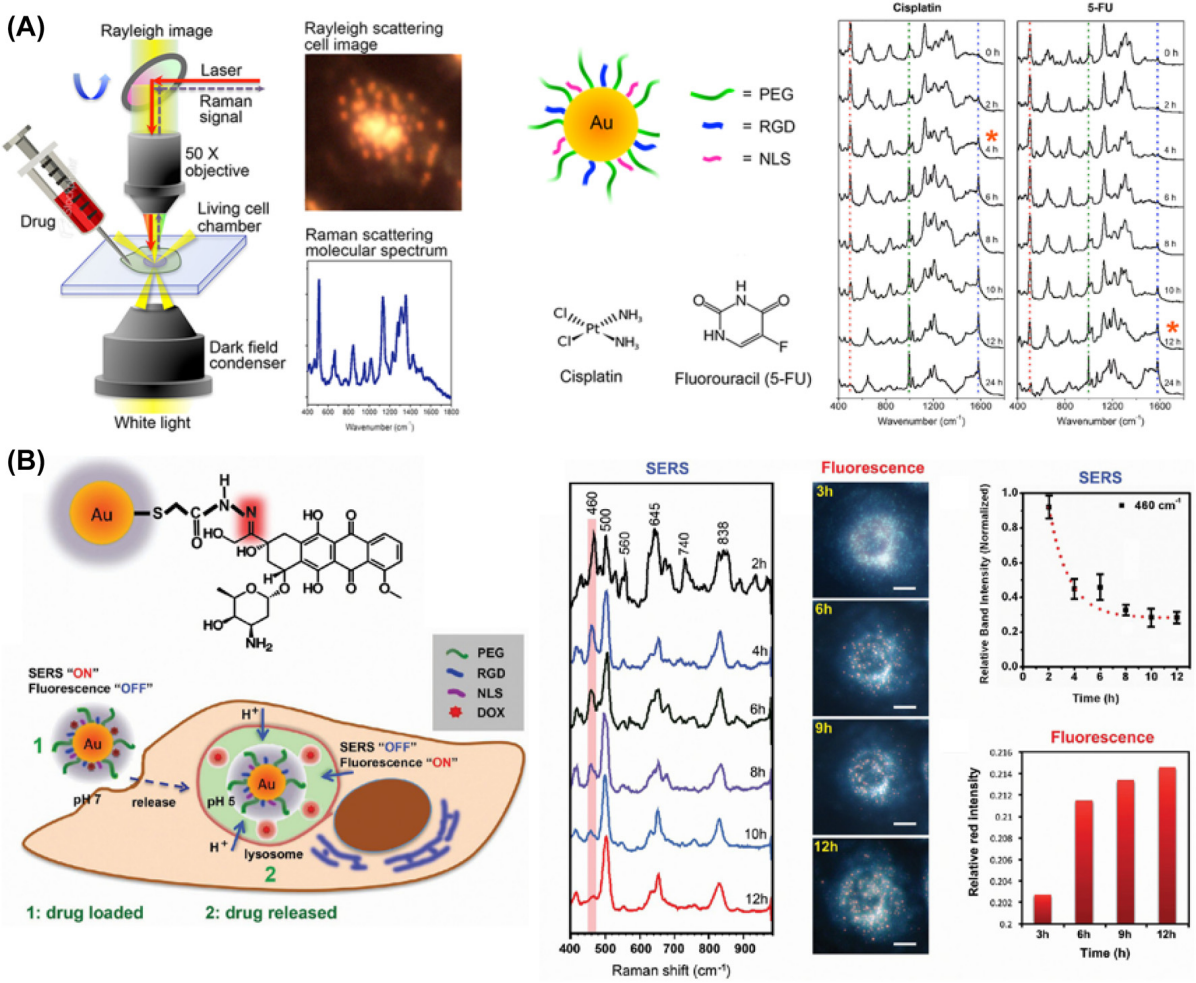
SERS in cell to monitor the dynamic changes. (A) Experimental setup of SERS and drug efficacy evaluation. (B) Conceptual description of DOX modified AuNPs and its use for SERS/fluorescence “on” and “off” and the results. Reprint permission was obtained for references [39], [40].

Austin et al. [39] demonstrated the potential of SERS-based cell imaging as a new tool for determining drug efficacy with nuclear targeting peptide-modified AuNPs (24 ± 3 nm) in Figure 2A [39]. In this work, the imaging quality of single cells is not greatly highlighted. However, it is possible to obtain a dose-dependent SERS response in real time from the nucleus in low concentrations of cisplatin or 5-FU. Interestingly, the decreased band at 500 cm^−1^, a disulfide bond of protein, was found to be the signal of cell death. It is possible to monitor the significantly different dynamics of drugs based on the SERS spectra. The same group reported, in 2013, that using AuNPs (28 ± 3 nm) conjugated with doxorubicin (DOX) enabled the observation of the dynamics of drug delivery in a single cell via Raman and fluorescence imaging. Based on the fluorescent property of DOX and a pH-sensitive hydrazone linkage, the time-dependent SERS “off” and fluorescence “on” signals could be utilized to monitor the release dynamics of DOX in real time, as shown in Figure 2B [40]. After 6 h, the fluorescence signal was observed by the hydrazone bond breakage in the acidic pH environment of the lysosomes (pH 5.0). Recently, Koike et al. demonstrated the performance of SERS to monitor drug uptake in real time quantitatively in live cells using AuNPs, alkyne-tagged cathepsin B inhibitor, and slit-scanning Raman microscopy [41]. The alkyne tags exhibited Raman signals in the silent region of a cell, which can avoid the signal overlapping with that of cellular components. Along with SERS of AuNPs in the lysosome, the slit-scanning method allowed us to obtain cell images with high temporal resolution in less than 1 min, which enabled us to monitor the cellular uptake process in real time. This is one of the significant technological progresses in this area and is expected to be a greatly useful methodology in future drug development and a tool for fundamental life science.

### 3.2 SERS in a live cell for intracellular signal changes

The capability to monitor an intracellular signal change is also an important aspect as a potential analytical tool for cell biology [4]. The microenvironment of cells is so dynamic and transient in maintaining the homeostasis of living organisms [42], [43]. For example, a balance of reactive oxygen species (ROS) and scavengers is critical and regulated by cellular redox potential. A slight dysregulation of balance can induce the onset of pathologies such as metabolic disease, cancer, and neurodegenerative disease [44]. Intracellular pH is also a critical parameter in maintaining cellular functions such as transporting substances across the cell membrane, metabolism, growth, division, apoptosis, etc. However, normal Raman scattering cannot be used to measure the transient changes of intracellular pH or to detect the ROS or proton (H^+^) because of the low concentration and lack of molecular signatures of these species [45]. Thus, it is inevitably required to use plasmonic nanoparticles combined with chemical components to indicate the changed pH or the presence of ROS.

**Figure 3:**
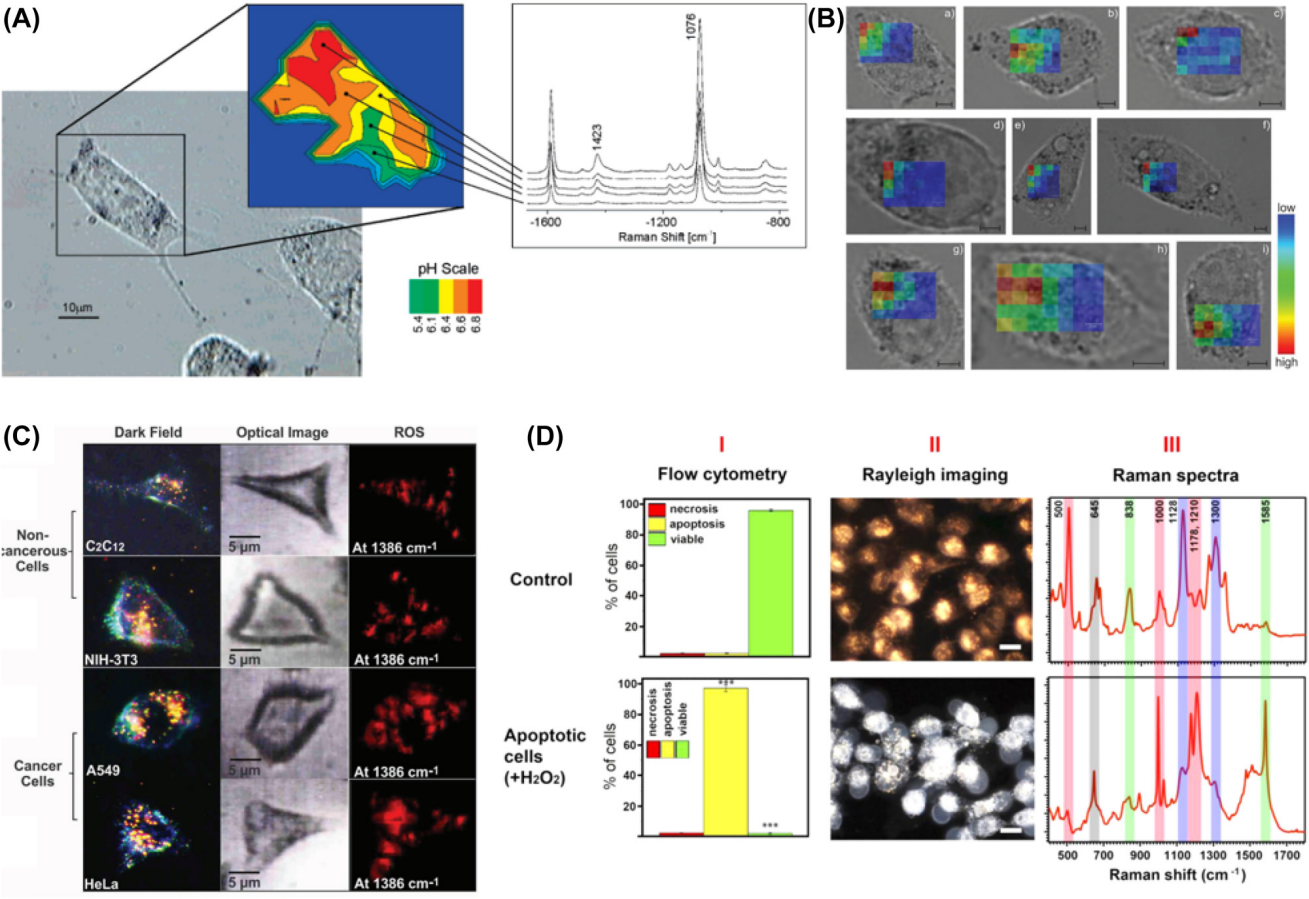
Method to monitor the changes of intracellular signals. (A) Photomicrograph of an NIH/3T3 cell and map of pH in the cell. (B) Bright-field images of J774 macrophage cells overlaid pH maps obtained from the AuNPs with (a–c) 2-NAT label, (d–f) 4-MBA label, and (g–i) both labels. (C) Dark-field, optical image, and SERS-based cell images in the live cancerous and noncancerous cells. (D) Viable and apoptotic cells flow cytometric analysis (I), dark-field images (II), and SERS spectra (III) of viable cells and apoptotic cells treated with 100 μM H_2_O_2_. Reprint permission was obtained for references [46], [47], [48], [49].

Kneipp et al. reported the capability of SERS in cells to obtain a map of pH in cells in a series of papers. They utilized 4-mercaptobenzoic acid (4-MBA) loaded gold nanoaggregates to obtain an image of pH in live cells [46]. The pH could be determined by comparing the SERS signal of two signature Raman shift at 1423 and 1076 cm^−1^ of 4-MBA as a function of pH. The ratio was varied between pH 6.0 and pH 10.0, which can be made pH map in a cell as shown in Figure 3A. Particularly, measuring pH below pH 6.0 is not feasible with SERS based on one-photon excitation, but they further expanded the measurable pH ranges down to pH 2.0 with two-photon excited SERS because of different selection rules of a molecule by two-photon excitation [46]. Furthermore, this two-photon–based SERS has created maps of pH in the macrophages containing plasmonic nanoparticles with 4-MBA (Figure 3B(a)–(c)), 2-naphthalene thiol (2-NAT) (Figure 3B(d)–(f)), and both (Figure 3B(g)–(i)) [47], [50]. Each pixel in a map represents a two-photon SERS spectrum. The chemical maps for pH were created with the integral of the ring stretching band at 1576 cm^−1^ of 4-MBA and the integrals of the bands at 1569, 1584, and 1624 cm^−1^ of 2-NAT. It is impossible to identify the distribution of the different reporters in the cell based on such a univariate analysis of the two-photon SERS spectrum. Under the individual example spectra discussed above, the chemical images indicate mainly 2-NAT SERS spectra in the perinuclear regions of the cells because of getting older lysosomal structures. The regions of the nuclei showed no detectable signals shown in Figure 3B due to the size restrictions of the nuclear pore, which did not allow the AuNPs to pass the nuclear membrane [47]. Recently, Zheng et al. reported using peptide-conjugated AuNPs to obtain a pH imaging of live cells during the cell cycle, comparing the performance with that of fluorescence at the same time [51].

Monitoring a reactive oxygen species in a cell could be achieved with a rationally designed ligand on AuNPs. Boronic derivatives have been frequently incorporated for this purpose. For example, Qu et al. demonstrated H_2_O_2_ production in live HeLa and HL-7702 cells using 4-carboxyphenylboronic acid-coated AuNPs. The detection limit was 80 nM [45]. The changes of aryl boronate to phenol in the presence of H_2_O_2_ lead to a decrease of the peak at 1562 cm^−1^ and the appearance of two new peaks at 1365 and 1600 cm^−1^.

Peng et al. and Chen et al. also demonstrated that sensing of H_2_O_2_ and peroxynitrite (ONOO–) was possible by the use of 4-mercaptophenylboronic ester-coated Au nanoparticles in a live cell [55], [56]. Distinguishing an oxygen species was possible by incorporating multiple reporter molecules for accurate identification. Cui et al. demonstrated the excellent multiplexing capability with a SERS nanoprobe by detecting five ROS species such as OH, H_2_O_2_, O_2_^−^, ROO⋅, and ^1^O_2_ [57]. Hemin protein and para-aminothiophenol (PTAP) were coated on AuNPs; hemin protein serves as a Fenton catalyst that can convert H_2_O_2_ into ^⋅^OH. The hydroxy radical induced a dimerization of PATP into 4,4-dimercaptoazobenzene, inducing new Raman active vibrational modes. Because of the selectivity and amenability of protein to subcellular targeting, incorporating protein was a useful approach for redox monitoring. Kumar et al. also reported the use of proteins such as myoglobin (Mb)-coated core–satellite type structures, where Mb protein could be acted as a ROS-responsive Raman reporter [48]. The presence of H_2_O_2_ triggered a conversion of six coordinated Fe(III)–OH2 (resting state) to Fe(IV)–O (Ferryl state), which was applied to discriminate the different amounts of ROS as shown in Figure 3C. Recently, Xiao et al. further advanced the method to quantify pH and H_2_O_2_ at the same time in real time with an Au shell structure coated with a silica layer saturated with 3-mercapto phenylboronic acid and 4-MBA as an H_2_O_2_ reporter and pH reporter, respectively [58]. This ratiometric SERS measurement enabled discrimination of the different pH and H_2_O_2_ amounts in healthy cells and unhealthy cells with or without oxidative stress.

Measuring the molecular dynamics during apoptosis or cell division is also an important task, which was demonstrated by Kang et al., as shown in Figure 3D. They demonstrated the use of nuclear-targeting AuNPs combined with dark-field microscopy to acquire SERS signal changes. The SERS signal could clearly be identifiable depending on the states of a cell during apoptosis [49] or cell cycling [59]. The changes of molecular signals from -S-S- vibration, DNA base, sugar-phosphate, and protein showed a characteristic pattern according to the cell cycle, such as G1, S, G2, and mitosis (M) phase [59].

**Figure 4:**
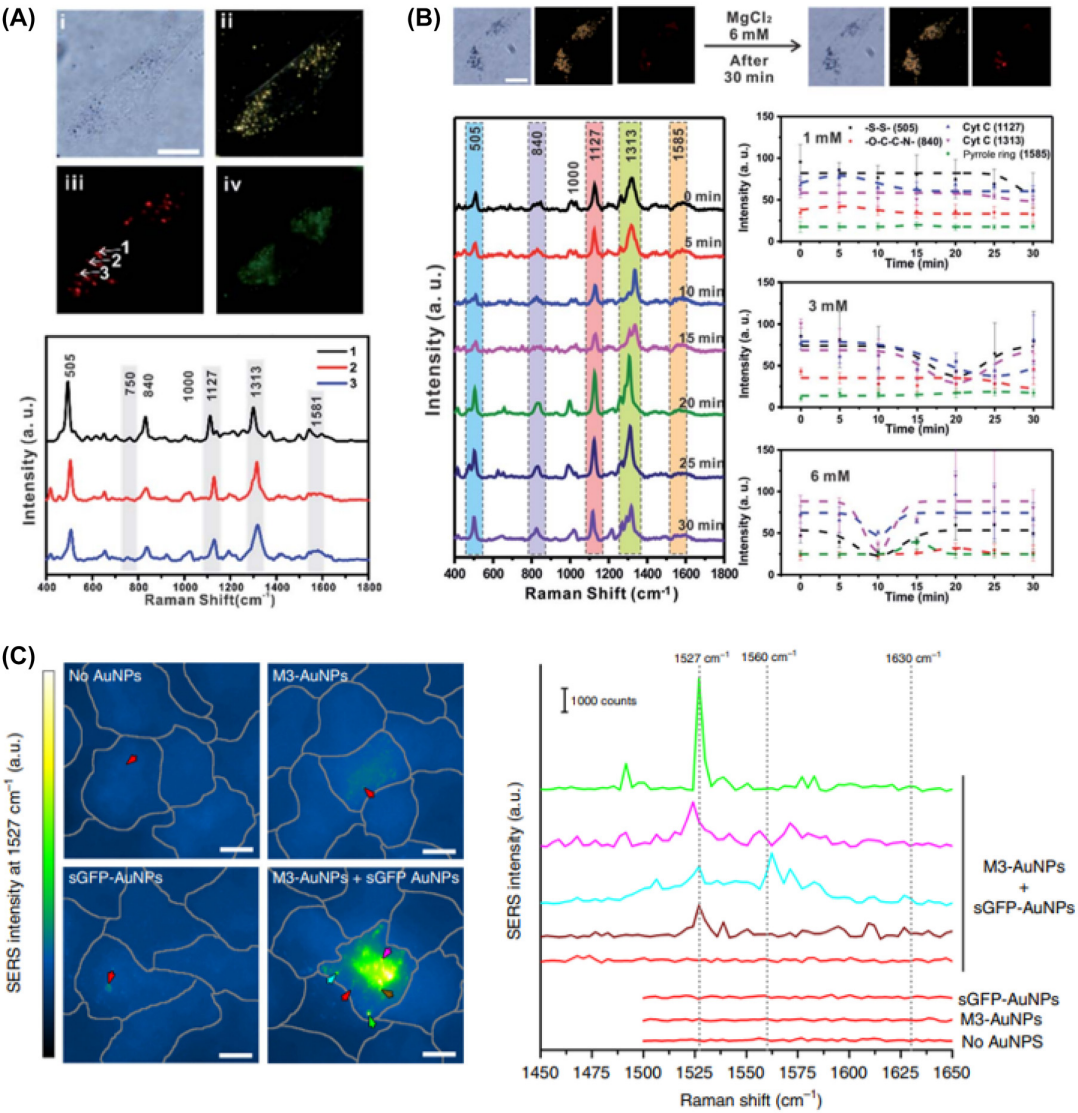
SERS in cell to monitor the changes of intracellular signals. (A) Bright-field (i), dark-field (ii), Raman image (iii), and fluorescence image (iv) of rheumatoid arthritis synovial fibroblasts incubated with TPP-AuNPs and Raman spectrum. (B) The changes in cell morphology and Raman spectrum by inducing membrane potential changes with MgCl_2_ (1 mM, 3 mM, and 6 mM). (C) SERS image of fixed cells at the GFP chromophore 1527 cm^−1^ imidazolinone/exocyclic C=C Raman mode. Reprint permission was obtained for references [52], [53], [54].

The identification of a specific single protein in a live cell will be a difficult task because of the presence of diverse proteins in a cell and their structural similarity between proteins. Among them, cytochrome C (Cyt-C) attracted great attention because of its important roles, such as cell death biomarkers and electron transfer in the mitochondria [60], [61]. In addition, Cyt-C has a unique structure called heme C consisting of a pyrrole ring and Fe ion, which can be a molecular signature for the Raman spectrum [60]. Shin et al. reported the use of triphenylphosphine-modified AuNPs for mitochondria targeting, which showed SERS signal of Cyt-C at 750, 1127, 1313, and 1581 cm^−1^ (Figure 4A). By inducing the mitochondrial membrane potential with a known chemical (MgCl_2_), the changed molecular signal in a time-dependent manner could be identified as shown in Figure 4B [52], [53]. Although no noticeable changes in cell images, the SERS spectrum in the mitochondria showed a noticeable change in intensity, shift, and peak splitting. Koker et al. demonstrated the cellular imaging of SERS in live cells by forming an assembly of AuNPs functionalized with split-fluorescent protein fragments. This is a direct confirmation of biomarker-assisted clustering of AuNPs in the cell membrane in Figure 4C [54].

In addition to monitoring the status of cells or cell cycle, identifying the status of stem cell differentiation is also an important field of application [62], [63]. Since stem cells have shown promise as candidates in diverse areas of cell therapy and regenerative medicine, the noninvasive method for accurate identification and discrimination of the differentiation of stem cells is greatly demanding. It is difficult to identify the differentiated cell types simply based on the morphology. Therefore, spectroscopic information with SERS can be a significant tool in this area. Huefner et al. demonstrated the successful identification of the differentiated neuronal cell types with nuclear targeting SERS probe and principal component analysis [63]. Milewska et al. demonstrated the use of gold nanoisland SERS substrate to monitor the differentiation of mesenchymal stromal cells [62]. In this work, they found the formation of calcium-phospholipid-phosphate complexes in the initial stage of osteogenic differentiation, and then the pronounced Raman signal of hydroxyapatite could be observed. The method of acquiring the SERS signal of a stem cell combined with chemometric data analysis could be further expanded to identify the cancerous stem cell or predict metastasis of cancer cells at a single cell level [64], [65], [66].

### 3.3 SERS in a live cell for advanced spatiotemporal resolution

Although there is a significant progress in SERS-based bioanalysis, the performance of spatiotemporal resolution could not compete with that of fluorescence. In the case of fluorescence, a single molecule-based experimental study is easy by utilizing a well-established optical setup such as a high-performance charge-coupled device (CCD), complementary metal-oxide-semiconductor camera, and a methodology to minimize the fluorescence background. It is possible to achieve wide-field cell imaging with video-rate temporal resolution (ms scale), which enables to monitor of transient changes in a biological event [67]. Furthermore, the super-resolution method enabled spatial resolution with several tens of nanometers in the fluorescence-based cell image. Typically, the super-resolution fluorescence image was obtained by inducing the “on–off” blinking and switching of single fluorophores [68], [69].

**Figure 5:**
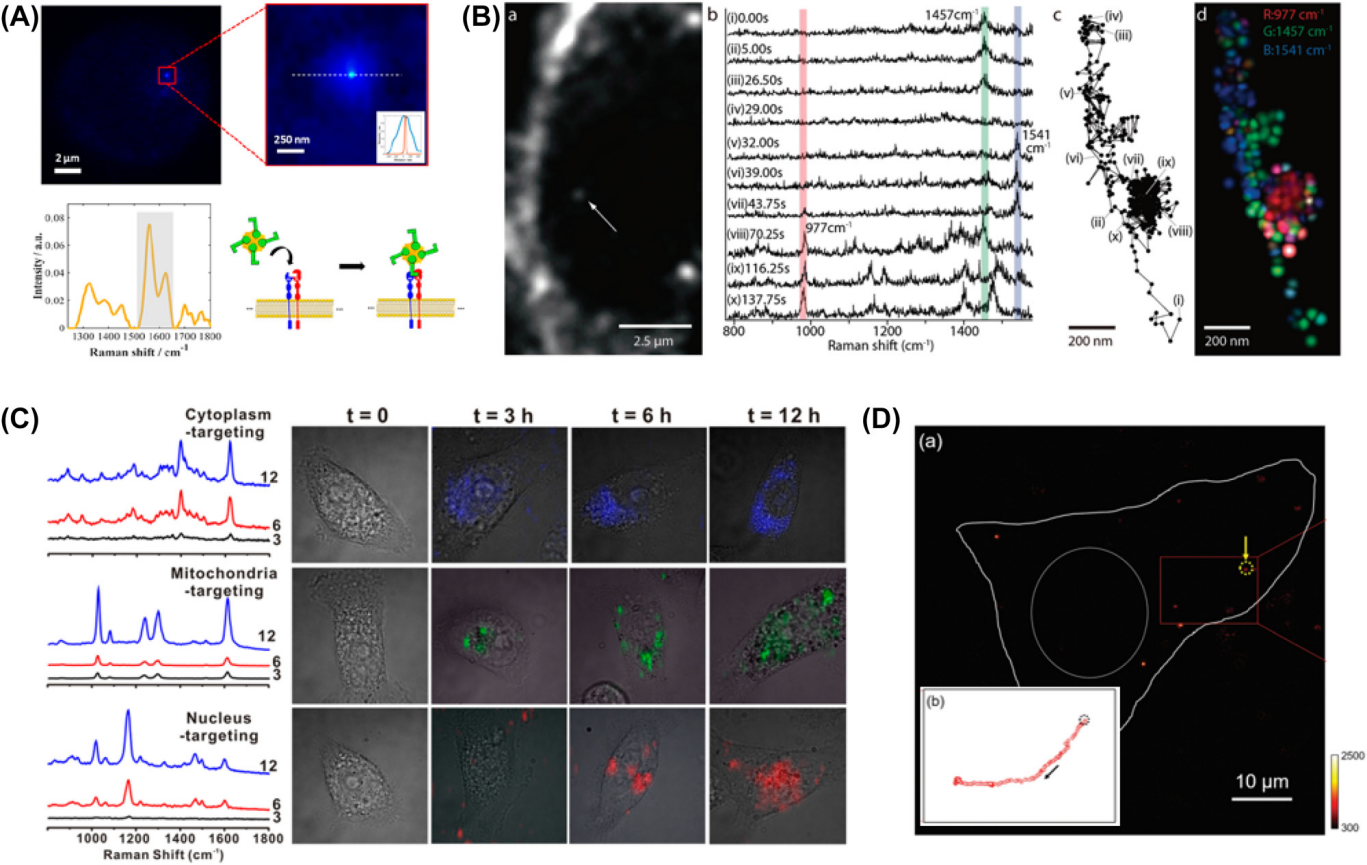
Improved spatiotemporal resolution of SERS in single cell. (A) Super-resolution SERS images of a single nanostar interacting with integrin receptor in membrane. (B) Dark-field image of single AuNPs in macrophage cell, SERS spectra nearby AuNPs, and the trajectory of AuNPs. (C) The spectra and cell images overlapped with SERS mapping images of organelle targeting Au-NNPs. (D) Single particle tracking of wide-field Raman imaging. Reprint permission was obtained for references [74], [75], [76], [77].

The super-resolution SERS for cell imaging also has been actively investigated. The natural time-dependent fluctuation of SERS signal in a single-molecule regime [70], SERS intensity fluctuations (SIFs) [71], can provide temporally fluctuating signals that are appropriate for localization algorithms such as stochastic optical reconstruction microscopy (STORM). Visiting the hotspots of molecules in solution is expected to be the possible origin of SIFs [70]. Therefore, the position (or centroid) of the emission can be determined within the diffraction-limited spot with a precision on the order of nanometers. Olson et al. reported the chemical imaging of collagen fibrils on a hexagonal silver nanohole array and bacteria with super-resolution SERS on silver substrates, which could be processed with STORM algorithms [72], [73]. For bacteria, the difference in chemical components in gram-positive or gram-negative bacteria could be imaged with high spatial resolution <50 nm [72]. Recently, Carlos et al. demonstrated super-resolution SERS for single particle imaging to track membrane receptors interacting with peptide-modified gold nanostars and the fixed cell on Ag island-covered surface. Using the temporal fluctuation of the Raman signal in the image and STORM methodology, a localization precision of less than 6.0 nm was reported as shown in Figure 5A [74].

Another example that made a significant advance in excellent temporal resolution is the work performed by Ando et al., shown in Figure 5B [75]. They reported the method to track the motion of 50 nm AuNPs in the dark-field image with a laser-beam steering mechanism synchronized with Raman spectroscopy, which provides a molecular map of organelle transport and lysosomal accumulation with 50 ms temporal resolution and 65 nm spatial resolution. It was possible to locate a single gold nanoparticle endocytosed by the cell. It allowed us to obtain a Raman spectrum from the moving nanoparticle for DNA phosphate at 977 cm^−1^, vibration mode of CH_2_ and CH_3_ at 1457, 1541 cm^−1^ common in lipids and proteins.

However, whole single-cell imaging with high spatiotemporal resolution was not feasible. Chen et al. reported the multiplexed cellular imaging with alkyne tags on Au and Ag core–shell probes [78]. Although it is successful for high-resolution SERS imaging of Hela cells, it takes 2 h for single-cell imaging with 1 s integration time per pixel (70 × 60 pixels) for 0.8 µm × 0.8 µm area. It should be noted that a minimized exposure time for each pixel is required to achieve a high-speed Raman image of a single cell because of an inverse trade-off between imaging quality and imaging speed. To address this limitation, it is required to use a highly bright SERS probe as a label. Kang et al. demonstrated the use of highly bright SERS probes and laser-beam splitting method to obtain a high-resolution SERS mapping in the live cell (Figure 5C). The laser beam splitting method can rapidly scan the area of interest much faster than that of a mechanical stage moving system. Kang et al. utilized an AuNPs (42 nm) with intra-nanogap that showed a detectable Raman response only with 10 ms exposure time [76], [79], [80]. With the Galvano mirror-based beam splitting method, high-resolution SERS mapping for a single cell was possible by acquiring 50 × 50 spectra from 38 µm × 38 µm area with 10 ms integration time per pixel. The total measurement time was about 27.5 s with a 1.0 ms CCD readout time. As shown in Figure 5C, high-quality single-cell images showing the distribution of AuNPs in the targeted subcellular organelles are presented.

Despite the advance in SERS-based cell imaging, the ultimate goal of cell imaging technology will be video-rate imaging performance [81]. In this regard, the scanning-based method has an intrinsic limitation for wide-area imaging [82]. Therefore, wide-field imaging technology is expected to be the possible solution to achieve video-rate imaging performance. Kim et al. further demonstrated the use of wide-field SERS imaging spectroscopy with high temporal resolution (200 ms) and high spatial resolution (512 × 512 pixels) for a wide area (100 µm × 100 µm) of a single cell in Figure 5D [77]. The significant advance in spatiotemporal resolution of this system enabled to track one or several particles of AuNPs with intra-nanogap structure in real time as well as monitoring the changes of molecular signals in the cell. Although this is not a perfect video-rate temporal resolution, it is sufficient to track particles inside a cell and monitor molecular signal transient changes.

### 3.4 SERS in a live cell by building in situ hot spots

**Figure 6:**
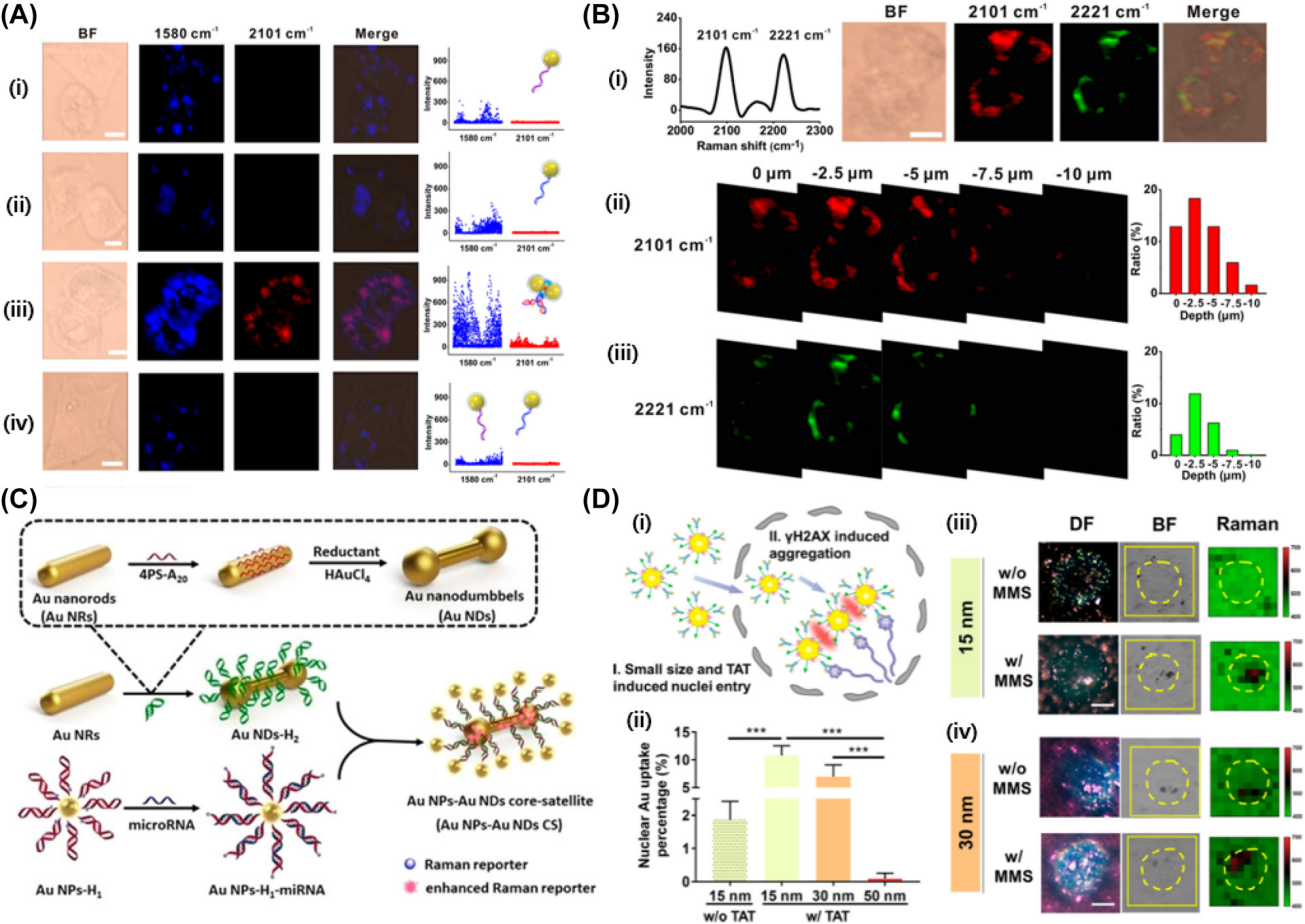
In-situ SERS imgaing with designed AuNPs in living cell. (A) Bright-field (BF), Raman mapping at 1580 cm^−1^ (blue channel) and 2101 cm^−1^ (red channel), merged images, and statistic Raman intensity for the cells treated with (i) probe 1, (ii) probe 2, and (iii) the mixture of probe 1 and probe 2. (iv) SERS imaging of miR-21 in living HEK-293 cells that were treated with probe 1 and probe 2. (B) Multiplexed imaging of miR-21 and miR-155 in living cells. (i) Raman spectra and cell images for –C≡C– and –C≡N–, 3D depth profile images with (ii) Raman peak at 2010 cm^−1^ and (iii) 2221 cm^−1^. (C) The fabrication route to Au nanodumbbell and AuNPs core-satellite structure, (D) intranuclear endocytosis of TAT and γH2AX antibody modified AuNPs, (i) γH2AX induced aggregation, (ii) uptake efficiency, dark field (DF), bright field (BF), and Raman images with (iii) AuNPs (15 nm) and (iv) AuNPs (30 nm) in the presence or absence of methyl methanesulfonate (MMS). Reprint permission was obtained for references [83], [84], [85].

It was well-known that the formation of hotspots in the nanoparticle aggregates is an important factor to obtain a greatly enhanced intensity of Raman scattering. However, the formation of hotspot, which is usually formed in a junction of dimer with a nanogap less than 1.0 nm, was achieved in solution or substrate, instead of live cell. It would be greatly beneficial, if the controlled formation of hotspot can be achieved in the live cell. Recently, Zhou et al. reported a strategy for *in situ* formation of hotspots in living cells [83]. In this work, the dimeric nanoparticles could be produced by the binding event between target miRNAs and locked nucleic acid sequences on the SERS probes, which enabled to obtain a Raman scattering with high specificity and reproducibility. In addition, they chose alkyne (–C≡C–) and nitrile (C≡N)-terminated molecules as a reporter because of strong and sharp single peaks in the cellular Raman-silent region (1800−2800 cm^−1^), where the intracellular endogenous biomolecules do not generate any Raman signals, thus eliminating background interference with target triggered nanoparticle dimerization. As shown in Figure 6A(i)–(iv), the distinctive Raman peak at 2101 cm^−1^ was appeared when probe 1 and 2 are present in MCF-7 cell with miR-21. However, the Raman peak was not observed in the HEK-293 cell, which is negative control for the expression of miR-21 (Figure 6A(iv)). They further demonstrated the capability in multiplexed imaging of diverse targets. The two different targets such as miR-21 and miR-155 could be simultaneously detected and imaged in a single cancer cell. As shown in Figure 6B(i), two peaks at 2105 cm^−1^ and 2221 cm^−1^ could be observed in the cellular silent region, corresponding to the –C≡C– and –C≡N– vibration of the probes. The 3D mapping images in Figure 6B(ii) and (iii) showed that the exogeneous Raman signal of –C≡C– and –C≡N– was mainly derived from the layer at a depth of 2.5–5 μm. Liu et al. further demonstrated that the “off-to-on” SERS strategies could detect miRAN expression in cell with plasmonic Au nanodumbbells and Au nanoparticles as satellites (Figure 6C) [84]. The target-triggered catalytic hairpin assembly induced the formation of core-satellite nanostructure, which showed an attomolar sensitivity for intracellular miRNA target. Very recently, Han et al. also demonstrated the formation of *in situ* hotspots in nuclei, in which gold nanoprobes with a small size (15 nm, 30 nm) modified with 4-mercapto-benzonitrile as Raman reporter molecules, TAT peptide for nuclear targeting, and γH2AX antibody for specific targeting [85]. As shown in Figure 6D, the AuNPs were successfully internalized in nuclei and γH2AX induced an aggregation of AuNPs and produced hot spots for a signal enhancement. The presence of methyl methanesulfonate (MMS) known to induce a DNA damage, followed by the local overexpression of γH2AX, which induce an aggregation of AuNPs. These recent papers showed the possible formation of hot spots in live cell by designing AuNPs with biomolecules for specific targeting. The SERS signal could be greatly amplified, in addition, the selection of SERS labels such as alkyne or nitrile played an important role in reducing the background signal of Raman scattering. It is expected that the highly bright signal intensity and molecular responsive properties of *in situ* hot spot formation envision the promising SERS-based single cell imaging technology for future bioanalytical applications.

## 4 Conclusions and outlook

Diverse research focus have been extensively made to overcome the fundamental limitations of Raman scattering (i.e., extremely low cross-section) as well as develop a new field of applications. In particular, the report on the possibility of single-molecule SERS in 1997 and the development of nanotechnology greatly expanded research interest in understanding the mechanism of single-molecule SERS and bioanalytical applications with SERS. Now, the fundamental mechanism of SERS is well understood. The methodologies for precisely controlled nanostructures to obtain a single molecule sensitivity are also relatively well established.

Currently, finding breakthrough applications that can address the limitation of fluorescence is a direction of SERS-based cell imaging technology. Since it has several advantages compared to conventional methods using fluorescence signals, research on applying Raman spectroscopy to cell biology is considered to be a natural procedure. Diverse approaches have been made to significantly improve the spatiotemporal resolution of Raman scattering for cell imaging. Applying SERS for cell imaging is a way to overcome the low signal intensity of normal Raman scattering, and the incorporation of new optical setups and nonlinear processes has been a direction of development [86], [87]. The temporal resolution is further improved by applying the line scanning method instead of the point scanning method [30], [61], [88], and it is expected that the method will be eventually advanced to obtain a molecular image of the entire single cell at a video rate (more than 20 images per second). Currently, a live single cell can be scanned within a few seconds, which was used to monitor the changes in molecular signals occurring within the cell. The intracellular movement process of nanoparticles with large Raman signal amplification can be monitored at a level approaching the video rate using wide-field microscopy.

The method of monitoring the changes of molecular information in a live cell is very useful for observing changes in various molecular signals occurring inside cells, especially in real time regarding the intracellular distribution of potential drugs, nucleic acid molecules, and other complexes inside cells. This may involve investigating apoptosis, cell division, and differentiation at the molecular level in real time. SERS also allows the tracking of individual particles inside a live cell and can learn information about molecular transport in organelles, accumulation in lysosomes, membrane protein diffusion, nucleus entry, rearrangement of the cell cytoskeleton, and more. Overall, cell imaging techniques based on intracellular SERS signals have great potential to become new research tools in cell biology [2], [3].

However, it is essential to send nanoparticles into cells to obtain SERS signals artificially, which is not perfect label-free technology. Additional improvement is being taken to minimize the possibility of distorting intracellular molecular signals by nanoparticles. The use of smaller size of nanoparticle and controlling an aggregation state by design in the target subcellular organelles are expected to be a desirable direction. Although there is a significant progress for super resolution SERS, further improvement for temporal resolution is also required to obtain an accurate molecular signals from a specific organelle within the live cell. The complex molecular signature in the Raman spectrum needs to be interpreted accurately to find out any meaningful information from a cell, for example, to find out metastatic cancer cells or differentiation of stem cells. For efficient data analysis, the field of chemometrics, which has already been used for an accurate analysis of complex spectroscopic data, is expected to be used extensively to find out valuable information. An accurate interpretation of multispectral data by incorporating machine learning and AI algorithm techniques in the future will result in a precise correlation between spectral data and biological phenomena.
